# A New Drill-Stock

**Published:** 1846-09

**Authors:** 


*A New Drill-Stock.*—We received from our correspondent, Dr. Jas. Robinson, of London, by the Caledonia, one of the most beautiful articles in the way of a drill-stock, which we have ever seen, and for which we beg to tender our most grateful acknowledgments. It is that described by the doctor in his recent work on the teeth, which we have noticed in another place in the present number of the Journal. He says, alluding to the manner of destroying the nerve in the root of a tooth, "For this purpose, Mr. McDowell, of Lincoln's Inn Fields, has invented a very ingenious and simple instrument, upon the principle of the helix lever. At the end of the screw is inserted a drill, which is made to revolve with the greatest rapidity, by means of a female screw attached and fixed in a handle. The mechanical parts oi the instrument are enclosed in a tube, and so arranged that it can be made equally available for excavating caries at almost any angle that may be required, by merely unscrewing the upper end of the tube, and substituting the additional heads, in which the action is reversed."—*Balt. Ed.*

